# Supplementary material for: Exceptional figure of merit achieved in boron-dispersed GeTe-based thermoelectric composites
Source: Nat Commun. 2024 Jul 14;15:5915. doi: 10.1038/s41467-024-50175-6 (PMC11246464; doi:10.1038/s41467-024-50175-6)
Supplement: Supplementary file 1 — Supplementary Information [file 41467_2024_50175_MOESM1_ESM.pdf]

Supplementary Materials

Exceptional figure of merit achieved in boron-dispersed GeTe-based thermoelectric composites

Yilin Jiang[1], Bin Su[1], Jincheng Yu[1], Zhanran Han[1], Haihua Hu[1], Hua-Lu Zhuang[1], Hezhang Li[1,2], Jinfeng Dong[1,3], Jing-Wei Li[1], Chao Wang[2], Zhen-Hua Ge[4], Jing Feng[4], Fu-Hua Sun[5], and Jing-Feng Li[1,4,5]*

[1] State Key Laboratory of New Ceramics and Fine Processing, School of Materials Science and Engineering, Tsinghua University, Beijing 100084, China

[2] Department of Precision Instrument, Tsinghua University, Beijing 100084, China.

[3] School of Materials Science and Engineering, Nanyang Technological University, Singapore 639798, Singapore.

[4] Southwest United Graduate School, Kunming 650092, China.

[5] Institute for Advanced Materials, Hubei Normal University, Huangshi 435002, China

*Correspondence and requests for materials should be addressed to J.-F.L. (email: jingfeng@mail.tsinghua.edu.cn)

Part I. Single Parabolic Band (SPB) modeling

According to the single parabolic band model[1,2], thermoelectric properties are given by

Seebeck coefficient

$$S=\frac{k_B}{e}\left(\frac{(\lambda+2)F_{\lambda+1}(\eta)}{(\lambda+1)F_{\lambda}(\eta)}-\eta\right) \tag{S1}$$

Hall carrier concentration

$$n_H=\frac{8\pi(2m_d^*k_BT)^{3/2}}{3h^3}\frac{1+\lambda}{1/2+2\lambda}\frac{F_\lambda^2}{F_{2\lambda-1/2}} \tag{S2}$$

in which

$$F_n(\eta)=\int_0^\infty\frac{\chi^n}{1+\mathrm{e}^{\chi-\eta}}\,\mathrm{d}\chi \tag{S3}$$

Electrical thermal conductivity is calculated according to the Wiedemann-Franz law[3],

$$\kappa_e=L\sigma T \tag{S4}$$

where L represents the Lorentz number. L is given by

$$L=\left(\frac{k_B}{e}\right)^2\left(\frac{(\lambda+3)F_{\lambda+2}(\eta)}{(\lambda+1)F_\lambda(\eta)}-\left[\frac{(\lambda+2)F_{\lambda+1}(\eta)}{(\lambda+1)F_\lambda(\eta)}\right]^2\right) \tag{S5}$$

where $k_{\rm B}$ is the Boltzmann constant, e is the electron charge, S is the Seebeck coefficient, $n_{\rm H}$ is the carrier concentration, and λ is the constant, respectively. λ is dependent on scattering factor r, which is equal to r+1/2. Assuming acoustic phonon scattering dominating the carrier scattering, r = – 1/2.

when the scattering factor taken into account,

Seebeck coefficient

$$S=[8\pi^2k_{\rm B}^2/(3eh^2)]m^*T\left[\pi/(3n)\right]^{2/3}(r+3/2) \tag{S6}$$

Part II. Debye-Callaway's model

Umklapp scattering process:

$$\tau_{\rm U}^{-1}=\frac{\hbar\gamma^2}{\overline{M}v_{\rm s}^2\theta_{\rm D}}\omega^2T\exp\left(-\frac{\theta_{\rm D}}{3T}\right) \tag{S7}$$

Normal process:

$$\tau_{\mathrm{N}}^{-1} = \beta \tau_{\mathrm{U}}^{-1} \tag{S8}$$

Grain boundaries scattering:

$$\tau_{\mathrm{B}}^{-1} = \frac{v_{\mathrm{s}}}{G} \tag{S9}$$

Point defects scattering:

$$\tau_{\mathrm{PD}}^{-1} = \frac{\overline{V}\omega^4}{4\pi v_{\mathrm{s}}^3}\Gamma \tag{S10}$$

Dislocation scattering[4,5]:

$$\tau_{\mathrm{ds}}^{-1} = \tau_{\mathrm{DC}}^{-1} + \tau_{\mathrm{DS}}^{-1} \tag{S11}$$

$$\tau_{\mathrm{DC}}^{-1} = N_{\mathrm{D}} \frac{V_a^{4/3}}{v_a^2}\omega^3 \tag{S12}$$

$$\tau_{\mathrm{DS}}^{-1} = 0.6 \cdot B_{\mathrm{D}}^2 N_{\mathrm{D}} \gamma^2 \omega \left[\frac{1}{2} + \frac{1}{24}\left(\frac{1-2\upsilon}{1-\upsilon}\right)\left(1+\sqrt{2}\left(\frac{v_{\mathrm{l}}}{v_{\mathrm{t}}}\right)^2\right)\right] \tag{S13}$$

Nano precipitates phonon scattering[5,6]:

$$\tau_{\mathrm{np}}^{-1} = \upsilon \left[\left(2\pi R^2\right)^{-1} + \left(\frac{4}{9}\pi R^2 \left(\frac{\Delta D}{D}\right)^2 \left(\frac{\omega R}{\upsilon}\right)^4 \right)^{-1} \right]^{-1} N_{\mathrm{p}} \tag{S14}$$

τ_{tot} is the total relaxation time, namely:

$$\tau_{\mathrm{tot}}^{-1} = \tau_{\mathrm{U}}^{-1} + \tau_{\mathrm{B}}^{-1} + \tau_{\mathrm{PD}}^{-1} + \tau_{\mathrm{DS}}^{-1} + \tau_{\mathrm{NP}}^{-1}. \tag{S15}$$

In the above equations, γ is the Grüneisen parameter, β is the ratio between normal process and Umklapp phonon scattering, υ is the Poison ratio, $\bar{V}$ is the average atomic volume, $\bar{M}$ is the average atomic mass, Γ is the point defect scattering parameter, d is the grain size, a is the lattice parameter, and N_{s} is the number of stacking faults crossing a line of unit length, B_{D} is Burgers' vector, N_{D} is the density of dislocations, R is the average radius for the precipitates D is the matrix density, ΔD is density difference between the precipitate and matrix, N_{p} is the number density of precipitates, A is the domain fitting parameter, d_{DB} is the average domain width respectively.

$\bar{V}$ and $\bar{M}$ referred to literatures[7], and γ and υ have been calculated by sound velocity v. N_{p}, N_{s}, N_{D} and d were measured via TEM characterization. D and ΔD were calculated

according to the theorical crystal structure.

Part III. Density functional theory (DFT) calculations

Density functional theory (DFT) calculations are performed using the plane-wave pseudopotential method in the Vienna Ab initio Simulation Package (VASP)[8–11]. The projector augmented wave (PAW) potentials are used to describe the interaction between electrons and ions. Generalized gradient approximation (GGA) in the scheme of Perdew–Burke–Ernzerhof (PBE) is employed to describe the exchange and correlation functions[12]. A 3×3×2 supercell based on the primitive cell of rhombohedral GeTe ($Ge_{18}Te_{18}$) and a supercell with one Ge atom replaced by Bi ($Ge_{17}BiTe_{18}$) are created to calculate band structure. A Monkhorst-Pack with 3×3×5 *k*-grid and cutoff energy of 450 eV are applied to represent the integration in the Brillouin zone of the electronic structure. Band-unfolding technique is utilized to unfold high-symmetry points to first Brillouin zone with PyVaspwfc tool[13], and the spin-orbit coupling (SOC) effect is considered throughout the band calculation with 0 magnetic moment of all atoms.

Part IV. Supplementary Data and Diagrams

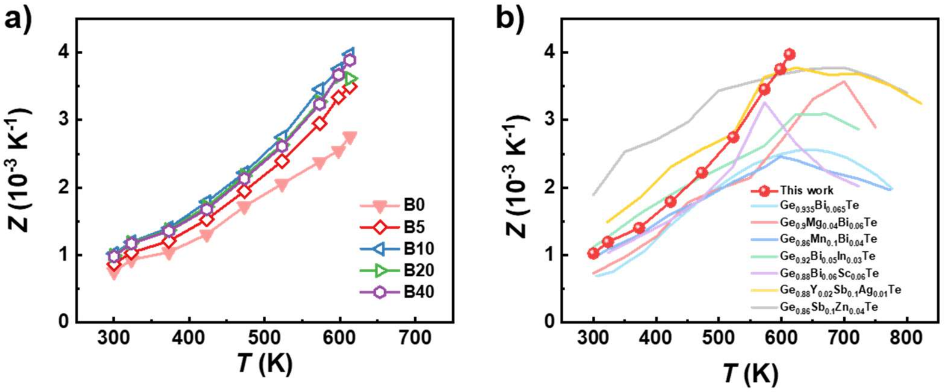

Supplementary Figure 1. *Z* value. (**a**) Temperature dependence of *Z* value for B/BGT samples. (**b**) The comparison in *Z* values between the BGT/B10 sample and the samples in literatures[14–20].

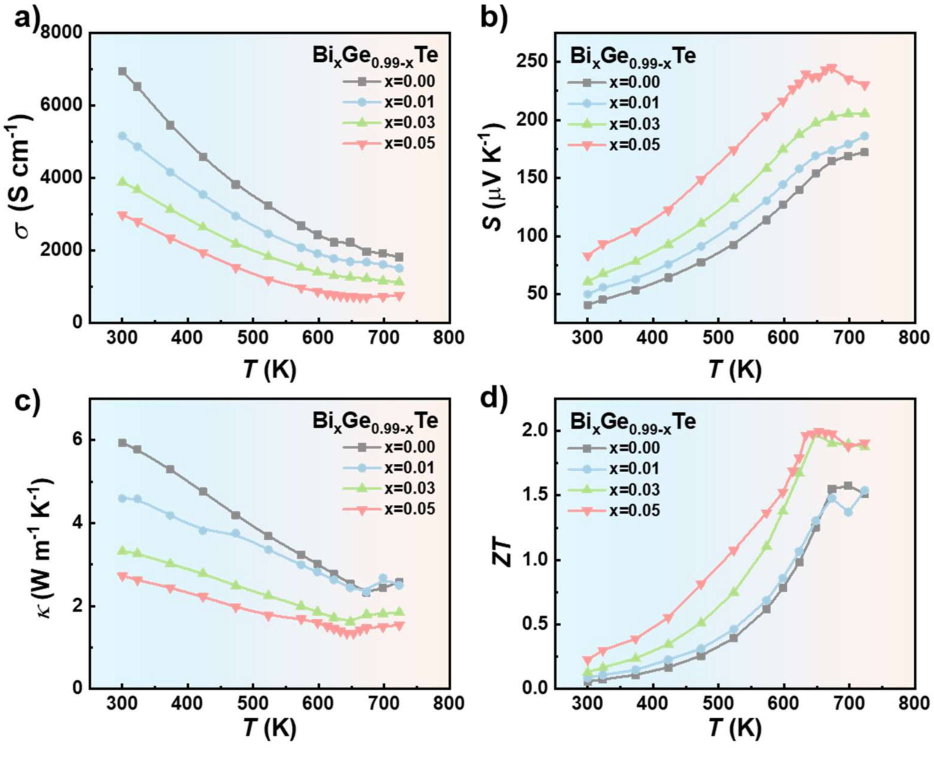

Supplementary Figure 2. Transport properties. The temperature-dependent electrical and thermal transport properties for Bi-doped samples.

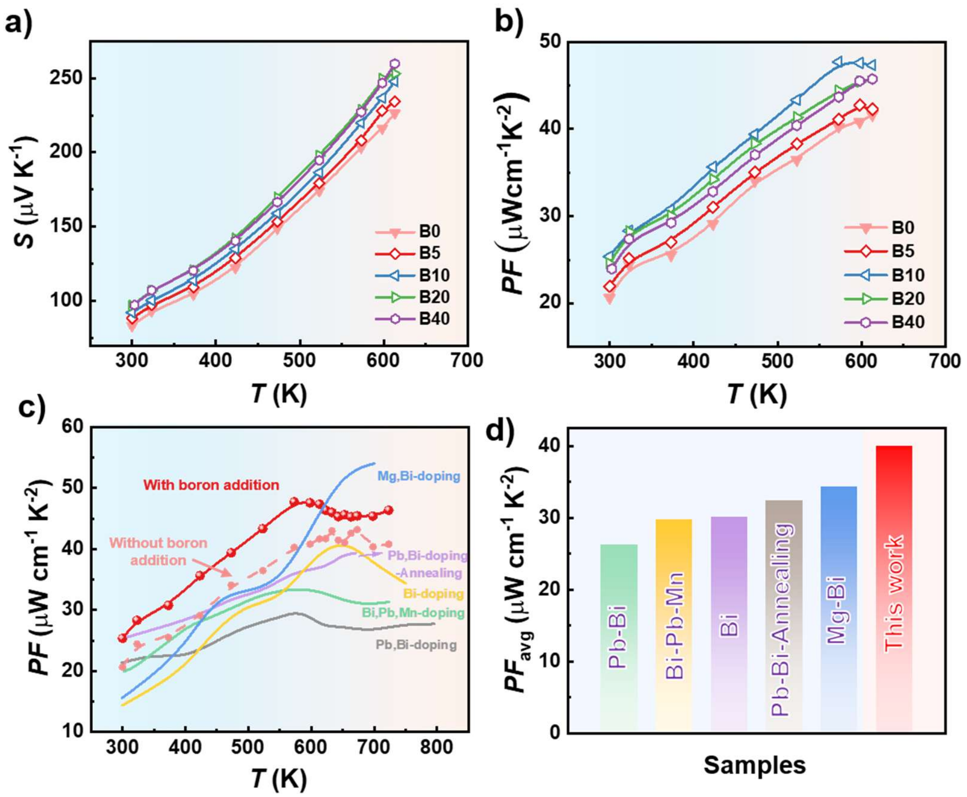

Supplementary Figure 3. Seebeck coefficient and power factor measurement. Temperature dependence of (**a**) Seebeck coefficient and (**b**) power factor for BGT/B samples. (**c**) The comparison in power factor and (**d**) average power factor between B/BGT samples (B0/BGT and B10/BGT samples) with the samples in literatures[14,15,21–23].

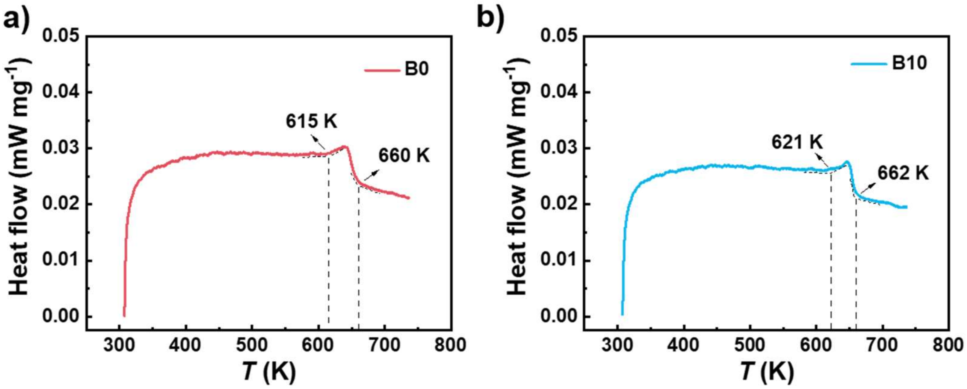

Supplementary Figure 4. DSC measurement. Differential scanning calorimetric (DSC) measurements performed on (**a**) B0/BGT and (**b**) B10/BGT samples.

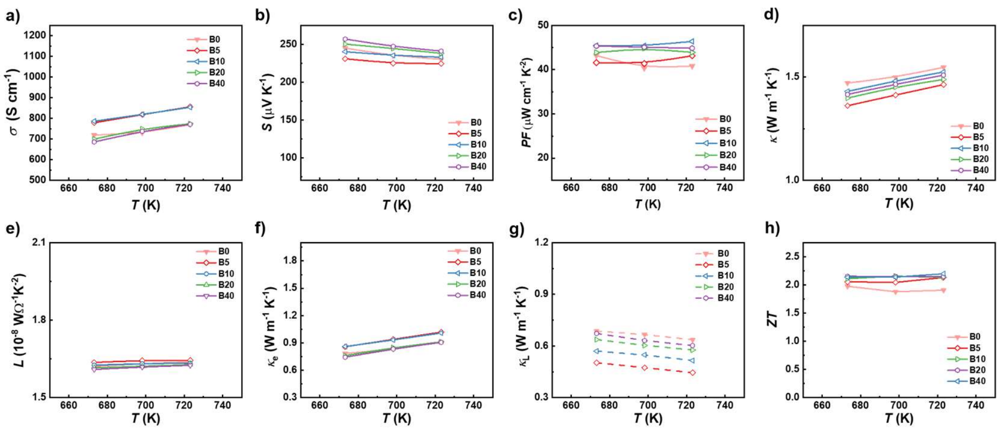

Supplementary Figure 5. The transport properties after phase transition. The temperature-dependent transport properties for B/BGT samples in C-GeTe.

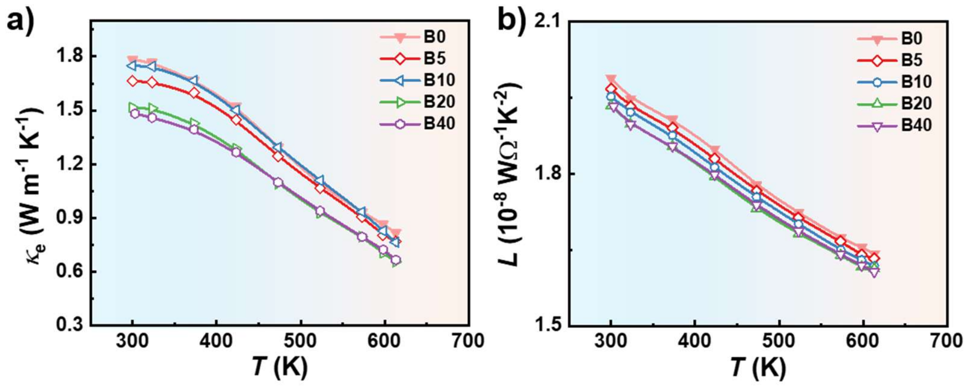

Supplementary Figure 6. Lorentz number and electrical thermal conductivity. (**a**) Calculated Lorentz number and (**b**) electrical thermal conductivity according to the SPB model and Wiedemann-Franz law, respectively.

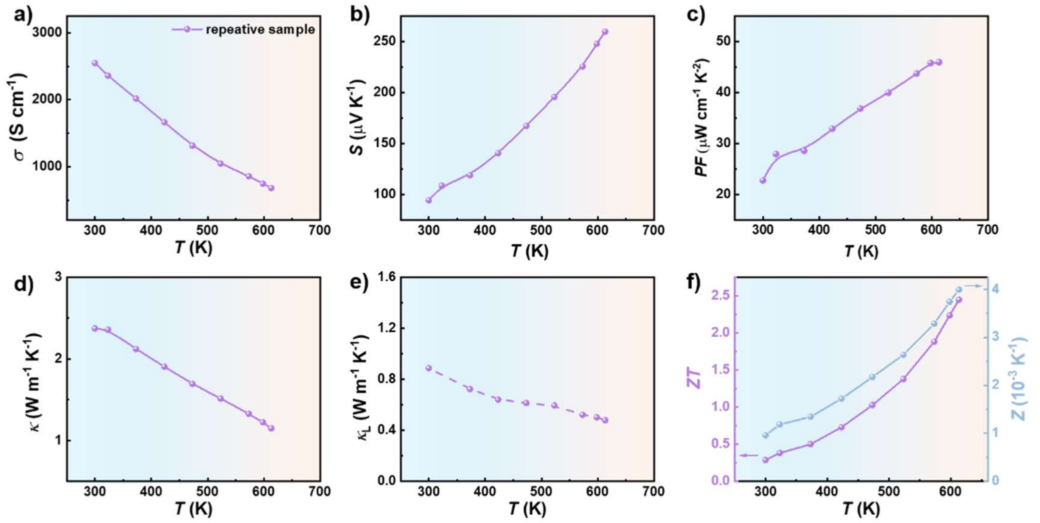

Supplementary Figure 7. TE performance for the repeated sample. The temperature-dependent transport properties for the repeated sample (B10/BGT).

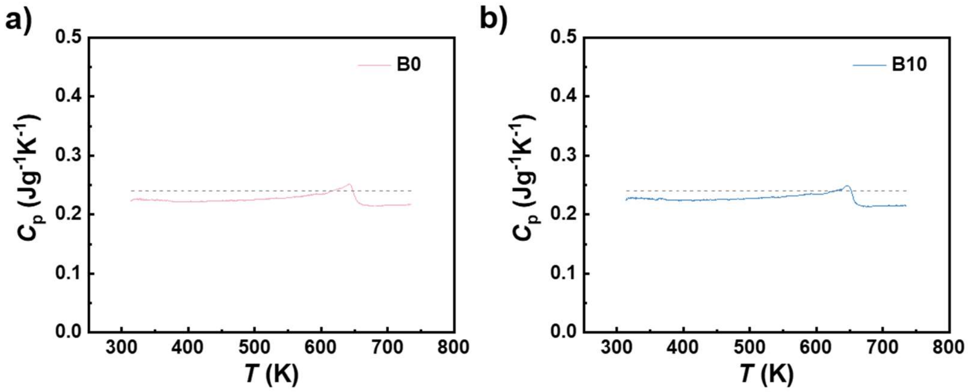

Supplementary Figure 8. **Specific heat capability measurement.** The C_p value for (**a**) the B0/BGT sample and (**b**) the B10/BGT sample.

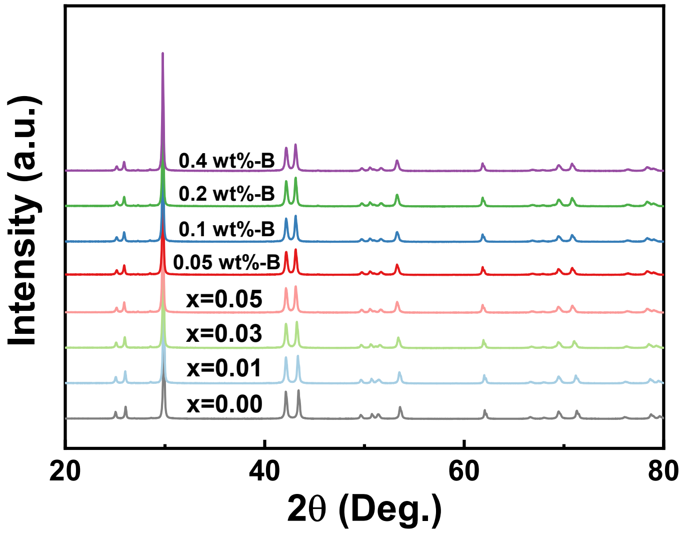

Supplementary Figure 9. Phase characterization. X-ray diffraction patterns of $Bi_xGe_{0.99-x}Te$-y wt. % B (x = 0.00, 0.01, 0.03, 0.05; y = 0.0, 0.05 0.10, 0.20, 0.40) samples.

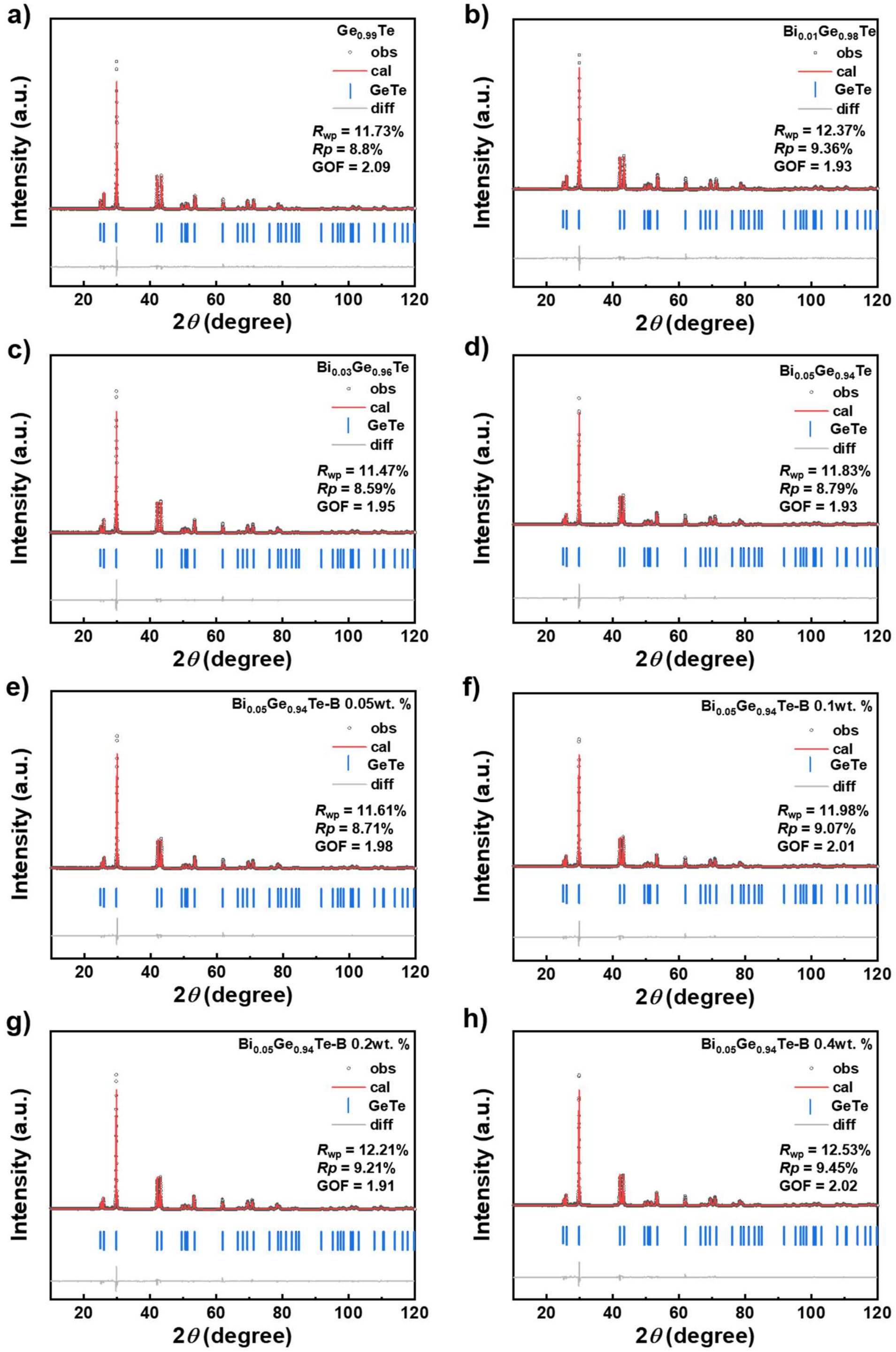

Supplementary Figure 10. Rietveld refinement. Rietveld refinement results for $Bi_xGe_{0.99-x}Te$-y wt. % B (x = 0.00, 0.01, 0.03, 0.05; y = 0.0, 0.1, 0.2, 0.4) samples.

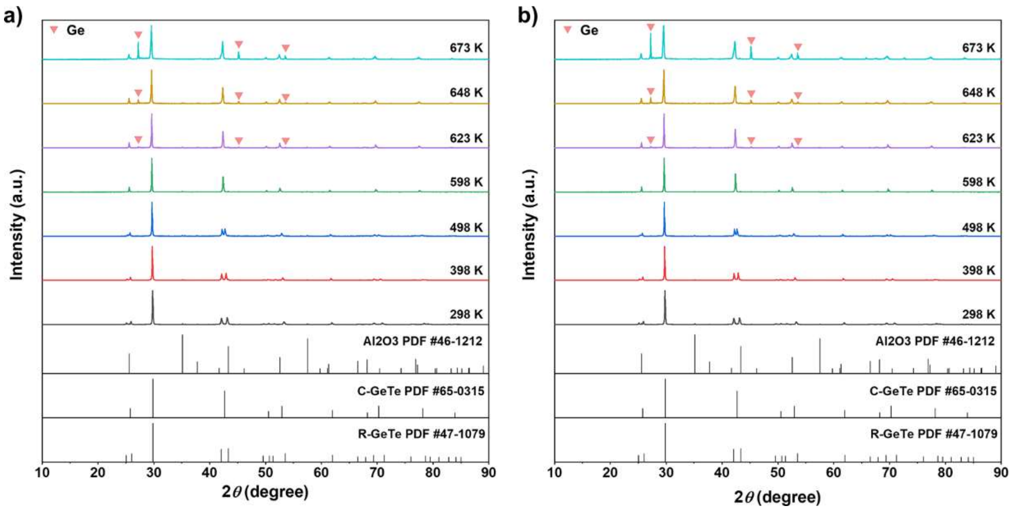

Supplementary Figure 11. Phase characterization in high temperatures. Temperature-dependent x-ray diffraction patterns of (**a**) BGT/B0 sample, (**b**) BGT/B10 sample. The sample holder is Al_2O_3 ceramic, and the edge of the holder can be detected by the x-ray. As a result, the peak of polycrystalline Al_2O_3 shown in the pattern. The Ge peaks are detected after 623 K, which is attributed to Ge precipitates generated in the high vacuum (10^{-4} - 10^{-5} mbar) during the high-temperature test.

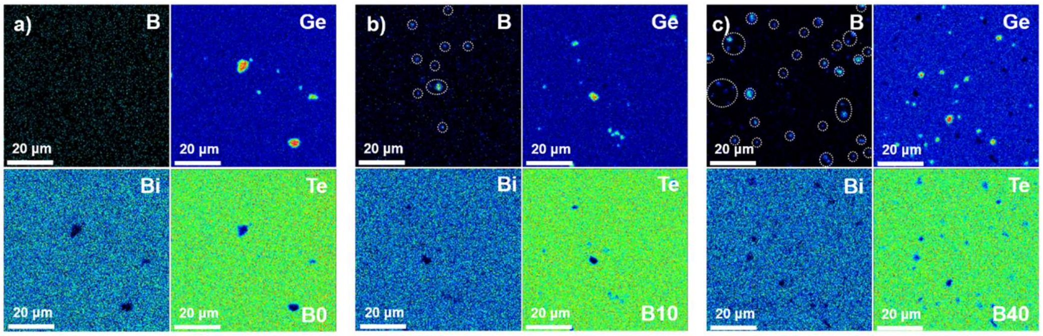

Supplementary Figure 12. Investigations on the element distribution. Electron probe micro-analyzer (EPMA) mapping images for (**a**) BGT/B0 sample, (**b**) BGT/B10 sample, (**c**) BGT/B40 sample.

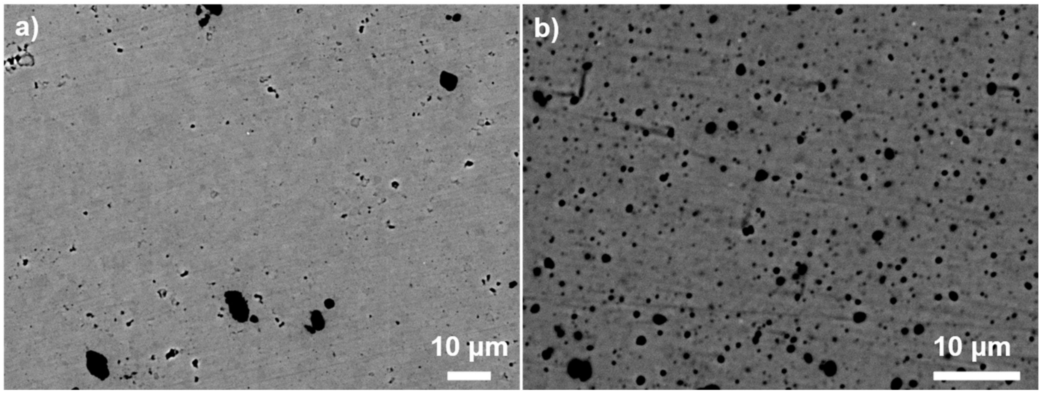

Supplementary Figure 13. **Back scattered electron imaging characterization.** The back scattered electron imaging for a) the B0/BGT sample and b) the B40/BGT sample.

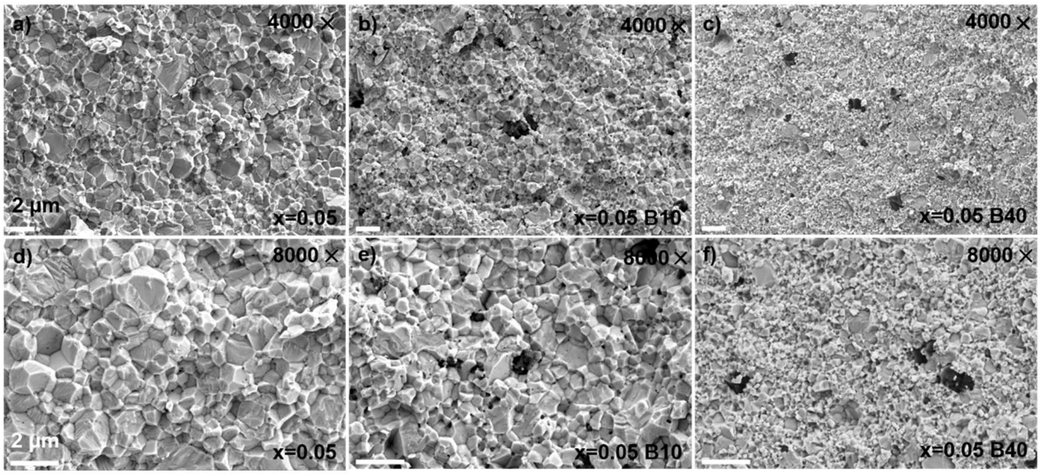

Supplementary Figure 14. Microstructure characterization. Scanning Electron Microscopy (SEM) images of surface fractured samples with different boron contents.

The crystal size decrease with increasing boron concentration.

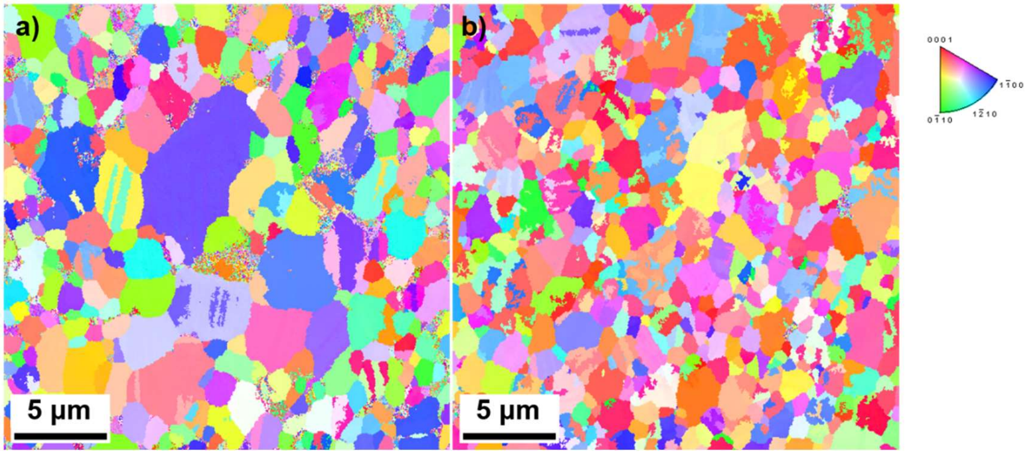

Supplementary Figure 15. Microstructure characterization. Electron backscatter diffraction (EBSD) images for (**a**) B0/BGT and (**b**) B40/BGT samples.

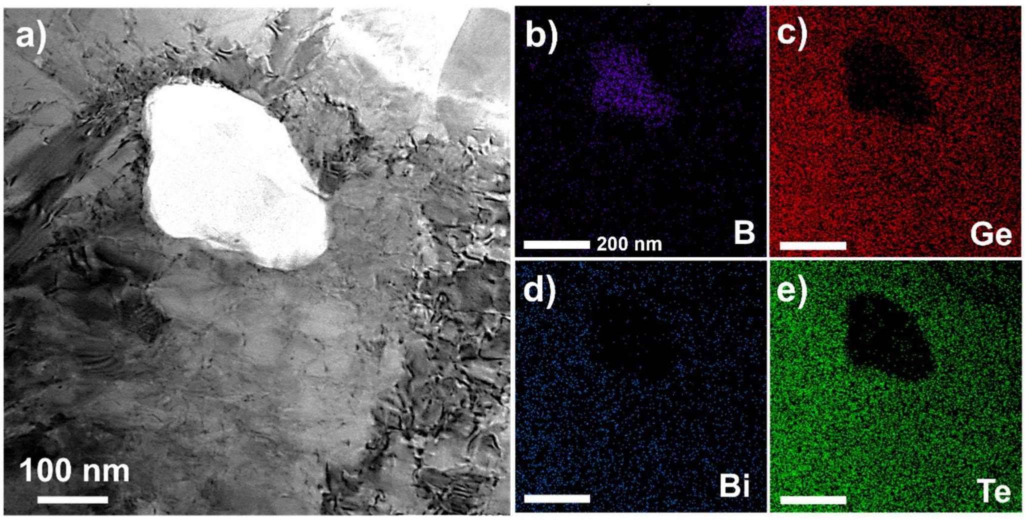

Supplementary Figure 16. Microstructure characterization. (**a**) The STEM image showing the boron inclusion for the B10/BGT sample, and (**b-e**) corresponding EDS mapping.

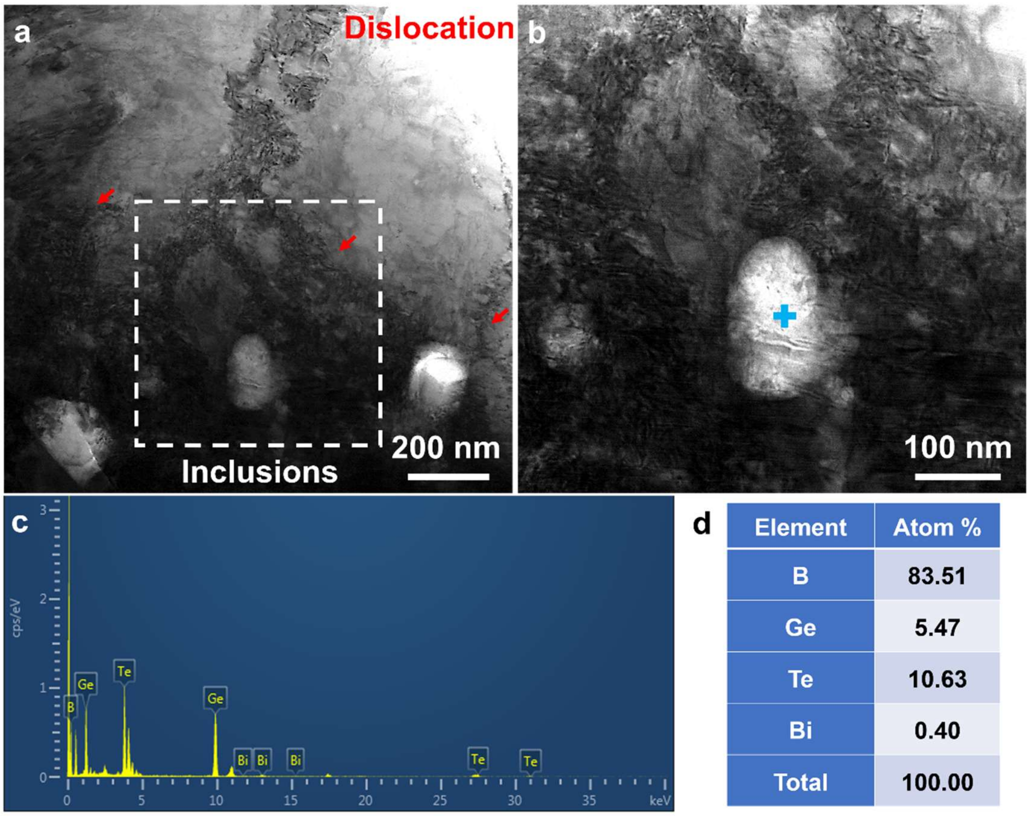

Element	Atom %
B	83.51
Ge	5.47
Te	10.63
Bi	0.40
Total	100.00

Supplementary Figure 17. Microstructure characterization. (**a**) STEM image showing the boron inclusion surrounded by dense dislocations for the B10/BGT sample. (**b**) The enlarged area for the white rectangle in (**a**) and (**c-f**) corresponding EDS mapping.

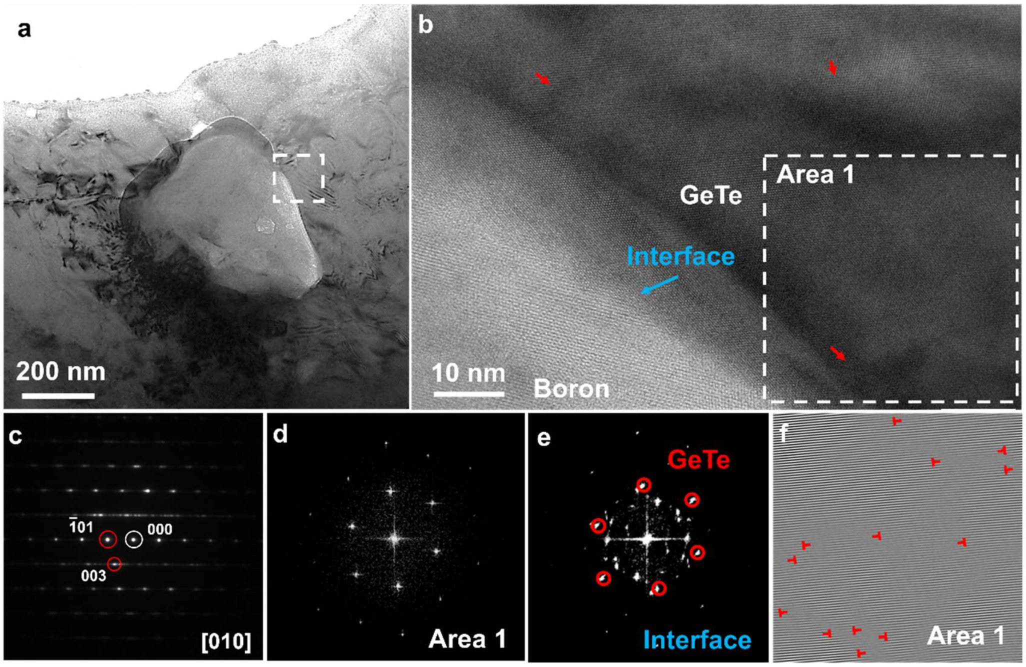

Supplementary Figure 18. Microstructure characterization. (**a**) The TEM image showing the morphology of the boron inclusion. (**b**) The HRTEM image showing the interface between the boron inclusion and GeTe matrix (the red arrows indicating the dislocations). (**c**) The SAED image of the boron inclusion. The corresponding (**d**) FFT and (**f**) IFFT images showing the area 1 in (**b**) indicating the dislocations in GeTe matrix. (**e**) FFT images at the interface.

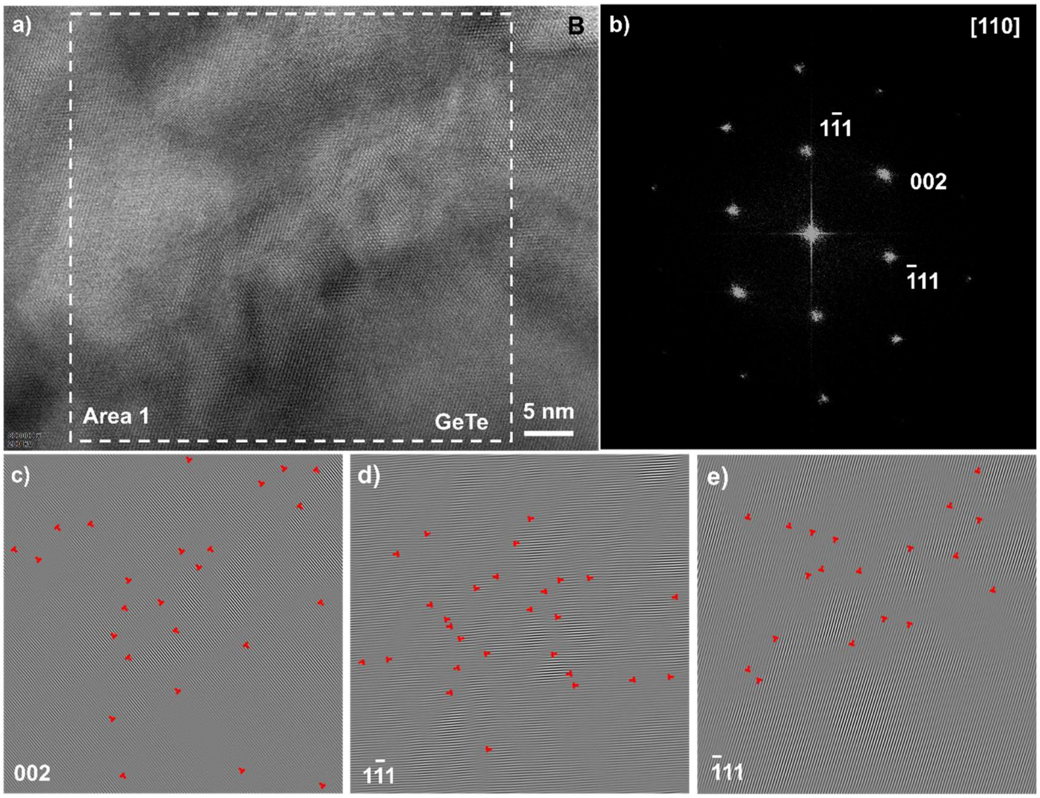

Supplementary Figure 19. Investigations on dislocations. (**a**) The HRTEM image of matrix near B/GeTe interfaces. The corresponding (**b**) FFT and (**c-e**) IFFT images showing the area 1 in (**a**).

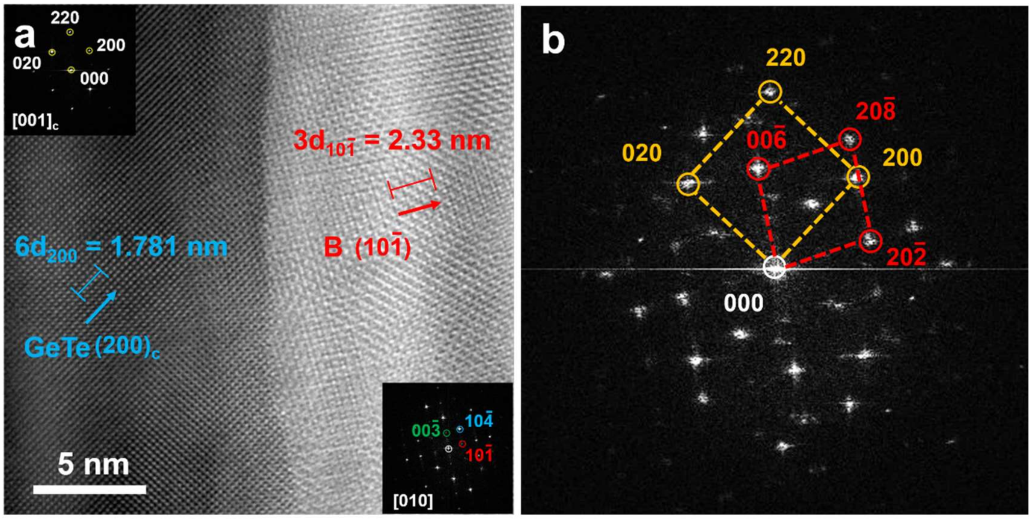

Supplementary Figure 20. Investigations on interfaces. (**a**) The HRTEM image showing one typical interface between the boron inclusion and GeTe matrix. (**b**) The corresponding FFT images indicating the incoherent interface.

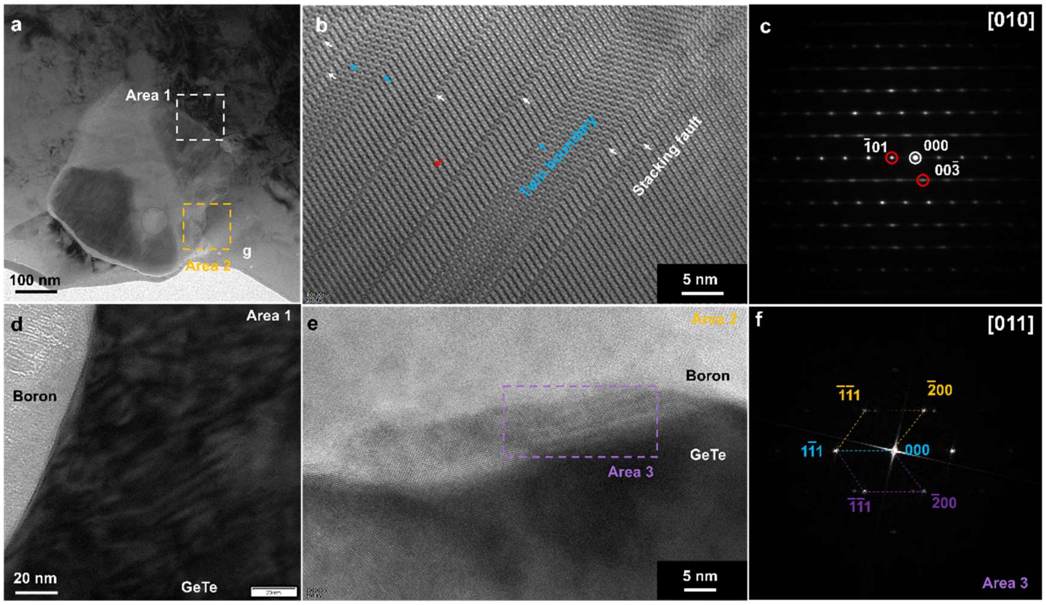

Supplementary Figure 21. Microstructure characterization. (**a**) TEM image of one boron inclusion. (**b**) HRTEM image and (**c**) SAED pattern of the boron inclusion. (**d**) The interface between boron and matrix with high-density dislocations. (**e**) HRTEM image showing the interfaces and the twin crystal for GeTe matrix. (**f**) FFT image for the twin crystal.

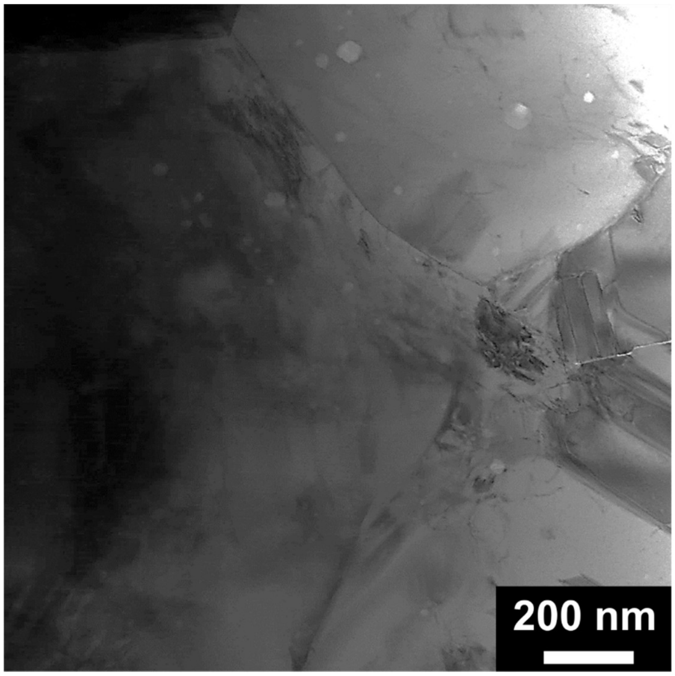

Supplementary Figure 22. Microstructure characterization. The STEM images showing the area without boron inclusion in the B10/BGT sample.

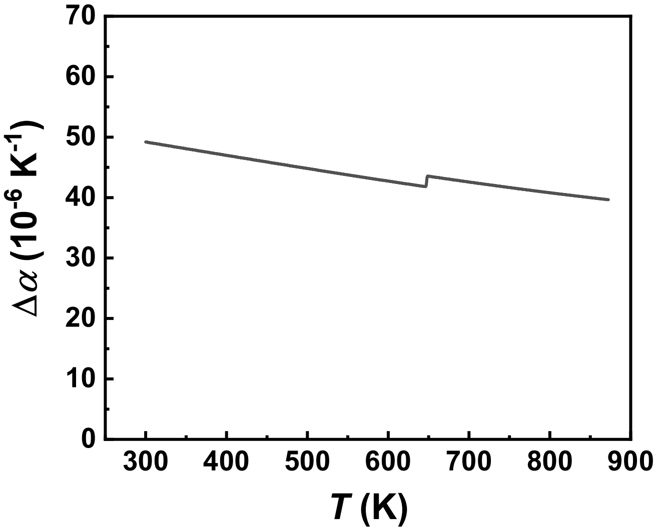

Supplementary Figure 23. Thermal expansion coefficient. The temperature-dependent TEC difference between GeTe and boron.

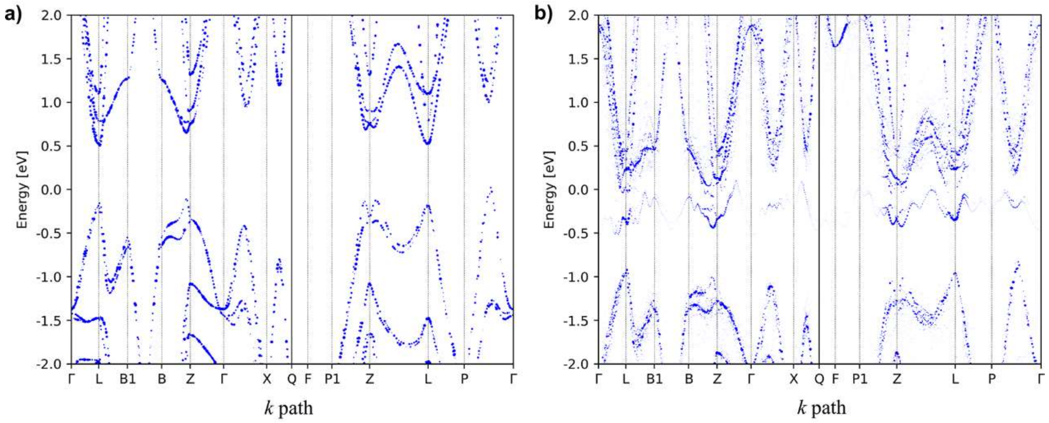

Supplementary Figure 24. Band structure. DFT calculated band structure for (**a**) Ge18Te18 and (**b**) BiGe17Te18.

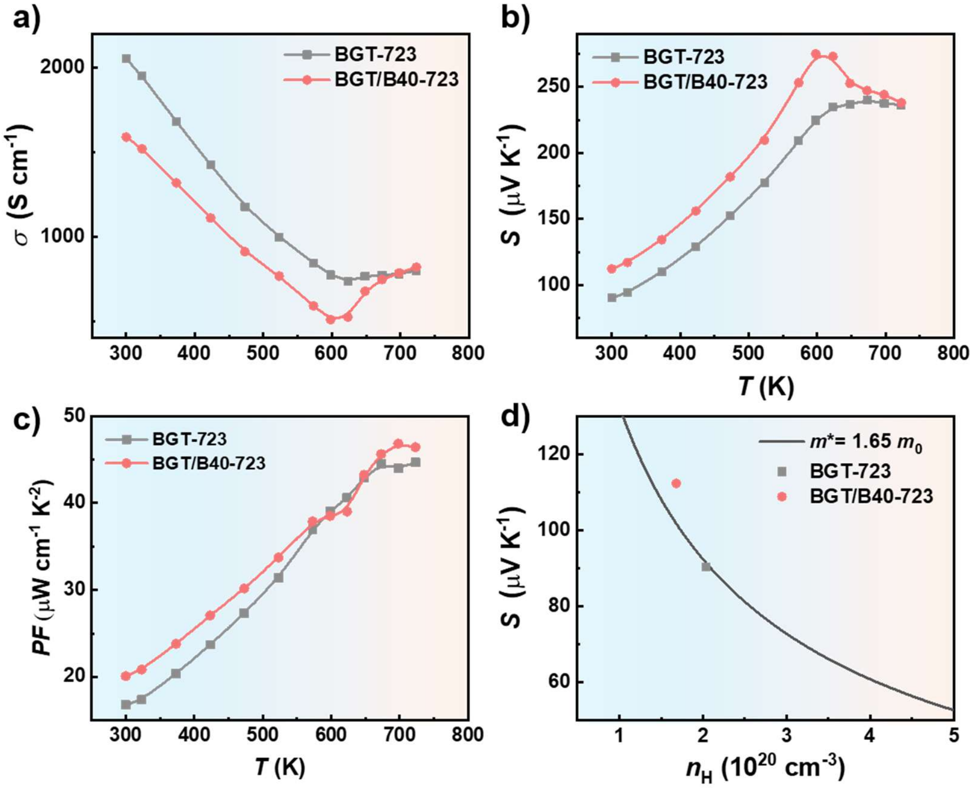

Supplementary Figure 25. Electrical transport properties of samples sintered at 723 K. The temperature-dependent electrical transport properties for the $Bi_{0.05}Ge_{0.94}Te$ and $Bi_{0.05}Ge_{0.94}Te$-0.4 wt. % B samples sintered at 723 K (BGT/B0-723, BGT/B40-723).

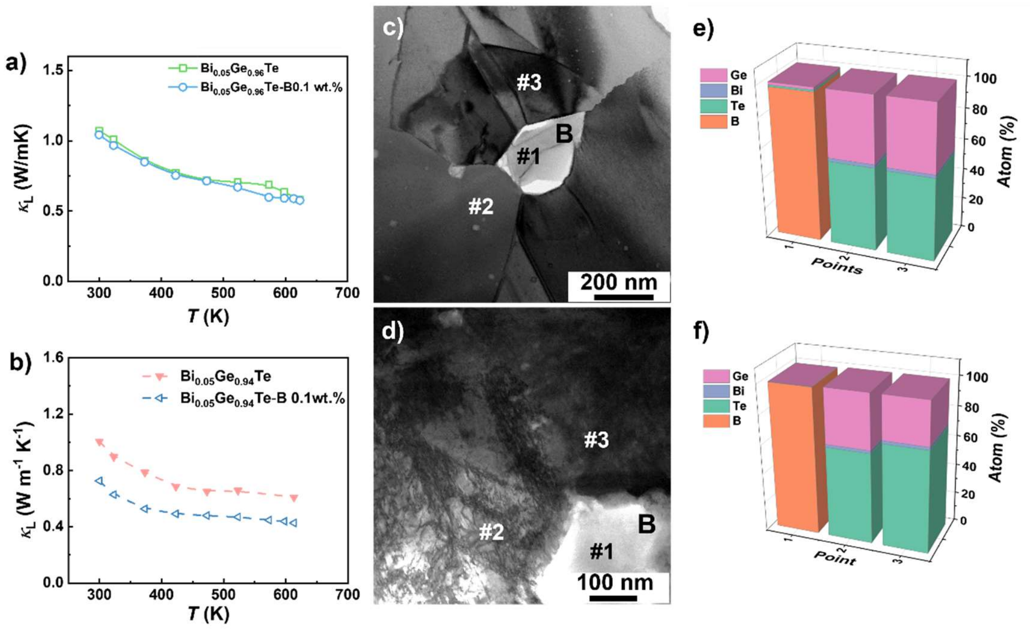

Supplementary Figure 26. The relationship of lattice thermal conductivity and microstructure. (**a**) The lattice thermal conductivity for $Bi_{0.05}Ge_{0.96}Te$ and $Bi_{0.05}Ge_{0.96}Te$-0.1 wt. % B samples, while (**b**) the lattice thermal conductivity for $Bi_{0.05}Ge_{0.94}Te$ and $Bi_{0.05}Ge_{0.94}Te$-0.1 wt. % B samples. (**c-d**) STEM images and (**e-f**) the corresponding atom contents for different points (#1, #2 and #3) for the $Bi_{0.05}Ge_{0.96}Te$-0.1 wt. % B sample and $Bi_{0.05}Ge_{0.94}Te$-0.1 wt. % B sample.

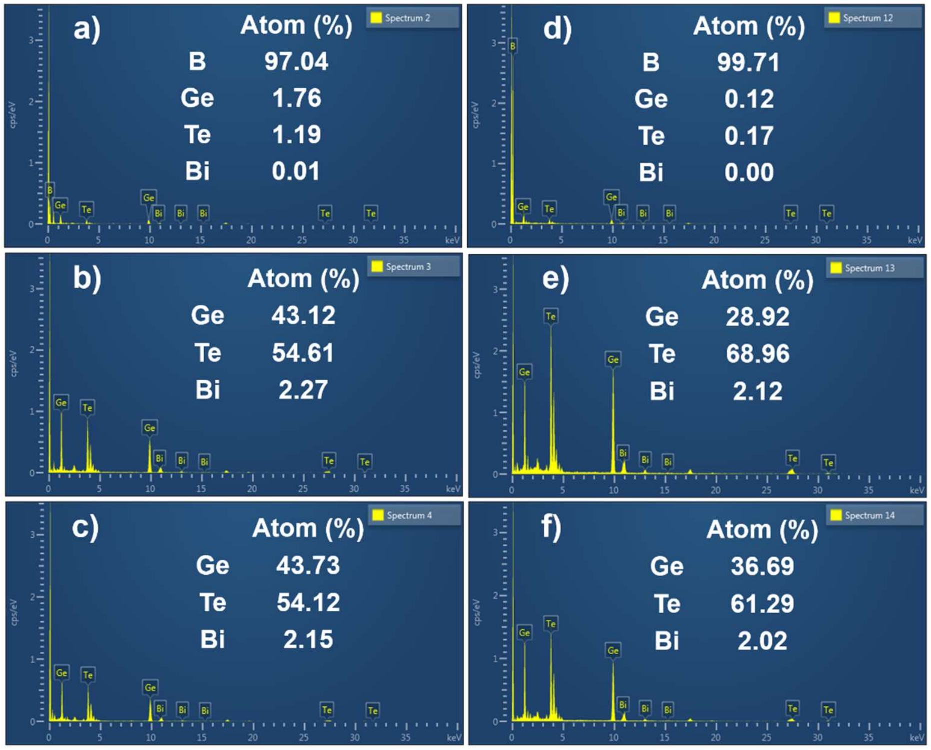

Supplementary Figure 27. Element contents. The corresponding atom contents of the different points (#1, #2 and #3) for (**a,b,c**) the $Bi_{0.05}Ge_{0.96}Te$-0.1 wt. % B sample and (**d,e,f**) $Bi_{0.05}Ge_{0.94}Te$-0.1 wt. % B sample in **Supplementary Figure 26**.

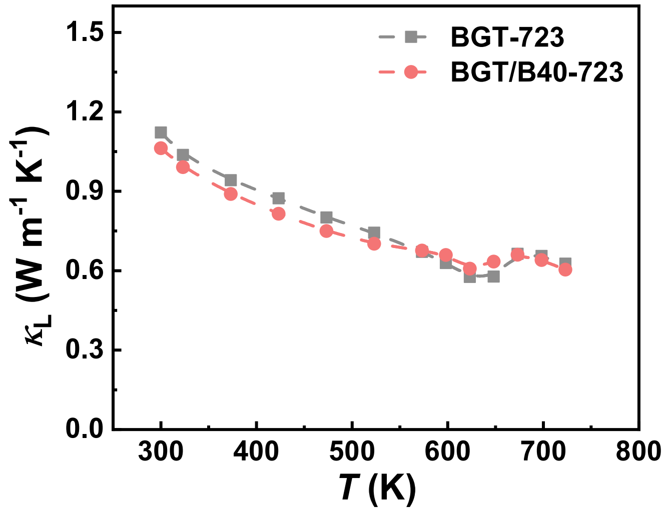

Supplementary Figure 28. Lattice thermal conductivity for samples sintered at 723 K. The temperature-dependent lattice thermal conductivity for the $Bi_{0.05}Ge_{0.94}Te$ and $Bi_{0.05}Ge_{0.94}Te$-0.4 wt. % B samples sintered at 723 K (BGT/B0-723, BGT/B40-723).

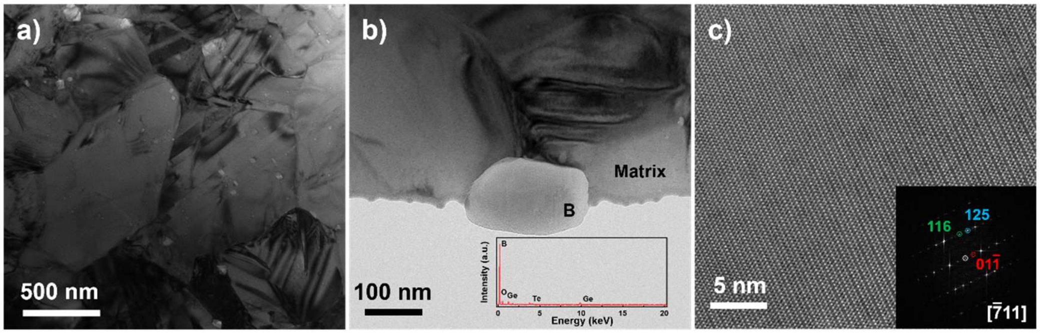

Supplementary Figure 29. Microstructure characterization for the sample sintered at 723 K. (**a**) Low magnification TEM image showing the microstructure of the $Bi_{0.05}Ge_{0.94}Te$-0.4 wt. % B sample sintered at 723 K. (**b**) TEM image of one boron inclusion, and the inset image showing the EDS results of the boron inclusion. (**c**) The HRTEM image and FFT result showing the structure of boron.

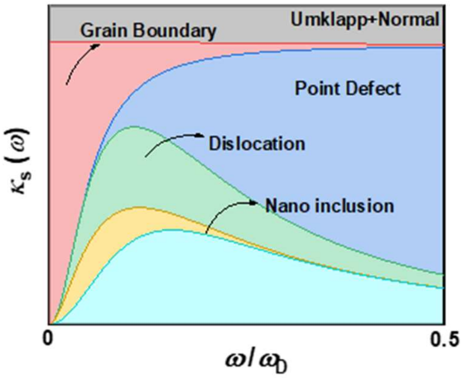

Supplementary Figure 30. Debye-Callaway model. Calculated κ_s using Debye-Callaway model with different phonon scatterings of Umklapp processes (U), grain boundaries (B), point defects (PD), dislocations (D) and precipitates (P) at 300 K.

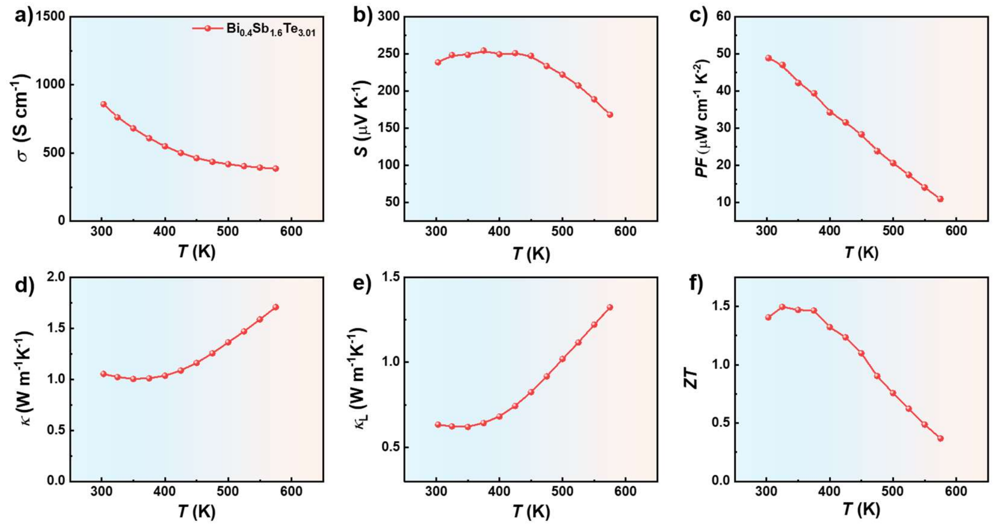

Supplementary Figure 31. TE performance for segmented single-leg device. The temperature-dependent transport properties for $Bi_{0.4}Sb_{1.6}Te_{3.01}$ TE materials.

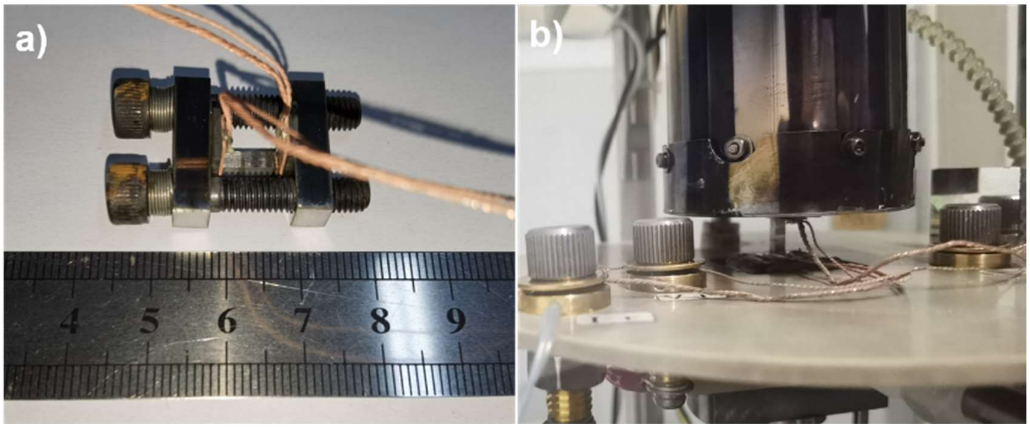

Supplementary Figure 32. TE device diagram. (**a**) The home-made soldering holder. (**b**) The Mini-PEM used to measure the conversion efficiency of a segmented single-leg.

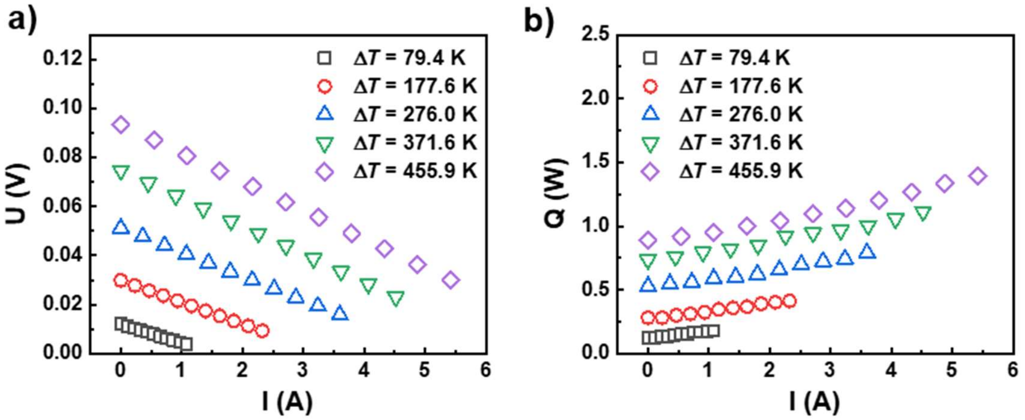

Supplementary Figure 33. Mini-PEM measurement. The tested voltage-current relationship and heat flow.

Supplementary Table 1. Transport parameters for $Bi_xGe_{0.99-x}Te$-B *y* wt.% samples at 300 K.

Sample	σ (10^3 S cm^{-1})	S (μV K^{-1})	n_H (10^{20} cm^{-3})	μ_H (cm^2 V^{-1} s^{-1})
**x=0.00**	6.94	40.77	6.20	69.9
**x=0.01**	5.68	47.17	5.11	69.3
**x=0.03**	3.89	60.97	3.85	63.1
**x=0.05**	2.99	83.14	2.60	71.7
**y=0.05**	2.82	88.25	2.50	70.4
**y=0.1**	2.99	92.17	2.61	71.4
**y=0.2**	2.61	96.94	2.47	66.1
**y=0.4**	2.53	97.25	2.43	65.0

Supplementary Table 2. Rietveld refinement parameters, doping dependent lattice parameters, unit cell volume, and the refinement agreement factors for $Bi_xGe_{0.99-x}Te$-B *y* wt.% samples at 300 K.

Sample	a (Å)	c (Å)	V (Å^3)	a_{cub} (Å)	α_{cub} (°)
**x=0.00**	4.16836(4)	10.66131(17)	160.425(4)	5.985	88.34
**x=0.01**	4.17566(4)	10.6529(2)	160.860(5)	5.988	88.43
**x=0.03**	4.18457(4)	10.6335(2)	161.253(4)	5.993	88.58
**x=0.05**	4.19555(5)	10.6176(2)	161.858(5)	6.000	88.74
**y=0.05**	4.19592(5)	10.6121(2)	161.802(5)	5.999	88.77
**y=0.1**	4.19604(6)	10.6124(3)	161.817(6)	5.999	88.77
**y=0.2**	4.19752(3)	10.6132(3)	161.943(6)	6.000	88.78
**y=0.4**	4.19728(5)	10.6115(3)	161.899(7)	6.000	88.78

Supplementary Table 3. Calculated scattering factor for BGT/B samples.

**Sample**	**Scattering factor (At 300 K)**
***y*=0**	-0.500
***y*=0.05**	-0.466
***y*=0.1**	-0.389
***y*=0.2**	-0.373
***y*=0.4**	-0.382

Supplementary Table 4. Parameters for phonon modeling studies

Parameters	Values
Debye temperature Θ_D (K)	209 (cal.)
Ratio of normal and Umklapp process β	2.5 (fitted)
Longitudinal sound velocity v_L (m/s)	3235 (exp.)
Transverse sound velocity v_T (m/s)	1910 (exp.)
Sound velocity v (m/s)	2116 (cal.)
Average atomic mass $\bar{M}$ (kg)	1.66×10^{-25}
Average atomic volume $\bar{V}$ (m^3)	2.708×10^{-29}
Grain size d (μm)	0.9 (exp.)
Point defect scattering parameter Γ	1.223 (cal.)
Grüneisen parameter γ	1.48 (cal.)
Lattice parameter a (Å)	6.0 (exp.)
Density of dislocation (cm^{-2})	1.11×10^{12} (exp.)
Magnitude of Burger's vector B_D (Å)	4.24 (ref.[24])
Density D (g cm^{-3})	6.16
Density difference between matrix and precipitates ΔD (g cm^{-3})	4.85
Number density of precipitates N_p (cm^{-3})	5.5×10^{11}

References

1 C.-L. Chen, H. Wang, Y.-Y. Chen, T. Day and G. J. Snyder, *J. Mater. Chem. A*, 2014, **2**, 11171–11176.

2 M. Hong, T. C. Chasapis, Z.-G. Chen, L. Yang, M. G. Kanatzidis, G. J. Snyder and J. Zou, *ACS Nano*, 2016, **10**, 4719–4727.

3 L.-D. Zhao, S.-H. Lo, J. He, H. Li, K. Biswas, J. Androulakis, C.-I. Wu, T. P. Hogan, D.-Y. Chung, V. P. Dravid and M. G. Kanatzidis, *J. Am. Chem. Soc.*, 2011, **133**, 20476–20487.

4 P. Carruthers, *Phys. Rev.*, 1959, **114**, 995–1001.

5 Z. Chen, Z. Jian, W. Li, Y. Chang, B. Ge, R. Hanus, J. Yang, Y. Chen, M. Huang, G. J. Snyder and Y. Pei, *Adv. Mater.*, 2017, **29**, 1606768.

6 N. Mingo, D. Hauser, N. P. Kobayashi, M. Plissonnier and A. Shakouri, *Nano Lett.*, 2009, **9**, 711–715.

7 M. Hong, Y. Wang, W. Liu, S. Matsumura, H. Wang, J. Zou and Z. Chen, *Adv. Energy Mater.*, 2018, **8**, 1801837.

8 G. Kresse and J. Hafner, *Phys. Rev. B*, 1993, **48**, 13115–13118.
9 G. Kresse and J. Furthmüller, *Comp. Mater. Sci.*, 1996, **6**, 15–50.
10 G. Kresse and J. Furthmüller, *Phys. Rev. B*, 1996, **54**, 11169–11186.
11 G. Kresse and D. Joubert, *Phys. Rev. B*, 1999, **59**, 1758–1775.
12 P. E. Blöchl, *Phys. Rev. B*, 1994, **50**, 17953–17979.
13 V. Popescu and A. Zunger, *Phys. Rev. B*, 2012, **85**, 085201.
14 J. Li, Z. Chen, X. Zhang, Y. Sun, J. Yang and Y. Pei, *NPG Asia Mater.*, 2017, **9**, e353–e353.
15 T. Xing, C. Zhu, Q. Song, H. Huang, J. Xiao, D. Ren, M. Shi, P. Qiu, X. Shi, F. Xu and L. Chen, *Adv. Mater.*, 2021, **33**, 2008773.
16 Z. Liu, J. Sun, J. Mao, H. Zhu, W. Ren, J. Zhou, Z. Wang, D. J. Singh, J. Sui, C.-W. Chu and Z. Ren, *Proc. Natl. Acad. Sci. USA*, 2018, **115**, 5332–5337.
17 S. Perumal, M. Samanta, T. Ghosh, U. S. Shenoy, A. K. Bohra, S. Bhattacharya, A. Singh, U. V. Waghmare and K. Biswas, *Joule*, 2019, **3**, 2565–2580.
18 Z. Liu, W. Gao, W. Zhang, N. Sato, Q. Guo and T. Mori, *Adv. Energy Mater.*, 2020, **10**, 2002588.
19 C. Liu, Z. Zhang, Y. Peng, F. Li, L. Miao, E. Nishibori, R. Chetty, X. Bai, R. Si, J. Gao, X. Wang, Y. Zhu, N. Wang, H. Wei and T. Mori, *Sci. Adv.*, 2023, **9**, eadh0713.
20 B. Jiang, W. Wang, S. Liu, Y. Wang, C. Wang, Y. Chen, L. Xie, M. Huang and J. He, *Science*, 2022, **377**, 208–213.
21 D.-Z. Wang, W.-D. Liu, M. Li, L.-C. Yin, H. Gao, Q. Sun, H. Wu, Y. Wang, X.-L. Shi, X. Yang, Q. Liu and Z.-G. Chen, *Chem. Eng. J.* 2022, **441**, 136131.
22 J. Li, X. Zhang, Z. Chen, S. Lin, W. Li, J. Shen, I. T. Witting, A. Faghaninia, Y. Chen, A. Jain, L. Chen, G. J. Snyder and Y. Pei, *Joule*, 2018, **2**, 976–987.
23 M. Zhang, Z. Gao, Q. Lou, Q. Zhu, J. Wang, Z. Han, C. Fu and T. Zhu, *Adv. Funct. Mater.*, **n/a**, 2307864.
24 Y. Jiang, J. Dong, H.-L. Zhuang, J. Yu, B. Su, H. Li, J. Pei, F.-H. Sun, M. Zhou, H. Hu, J.-W. Li, Z. Han, B.-P. Zhang, T. Mori and J.-F. Li, *Nat. Commun.*, 2022, **13**, 6087.
